# Supplementary material for: Cyclic Photoisomerization of Azobenzene in Atomistic Simulations: Modeling the Effect of Light on Columnar Aggregates of Azo Stars
Source: Molecules. 2021 Dec 18;26(24):7674. doi: 10.3390/molecules26247674 (PMC8709326; doi:10.3390/molecules26247674)
Supplement: Supplementary file 1 [file molecules-26-07674-s001.zip › molecules-1511738-supplementary.pdf]

Supplementary Material for "Cyclic Photoisomerization of Azobenzene in Atomistic Simulations: Modeling the Effect of Light on Columnar Aggregates of Azo Stars"

Markus Koch,[*,†] Marina Saphiannikova,[†,‡] and Olga Guskova[*,†,‡]

†*Institute Theory of Polymers, Leibniz Institute of Polymer Research Dresden, Hohe Str. 6, 01069 Dresden, Germany*

‡*Dresden Center for Computational Materials Science (DCMS), Technische Universität Dresden, 01062 Dresden, Germany*

E-mail: koch-markus@ipfdd.de; guskova@ipfdd.de

Contents

S1 Additional Figures **3**

S2 Mathematical Model of the Isomer Interconversion **7**

S2.1 *Trans* and *Cis* Isomers of Azobenzene 7

S2.2 Isomers of *TrisAzo* 8

S3 Descriptors of the Cluster Structure **10**

S3.1 Definition of Observables and Order Parameters 10

S3.2 Defect Detection Algorithm 11

References **12**

S1 Additional Figures

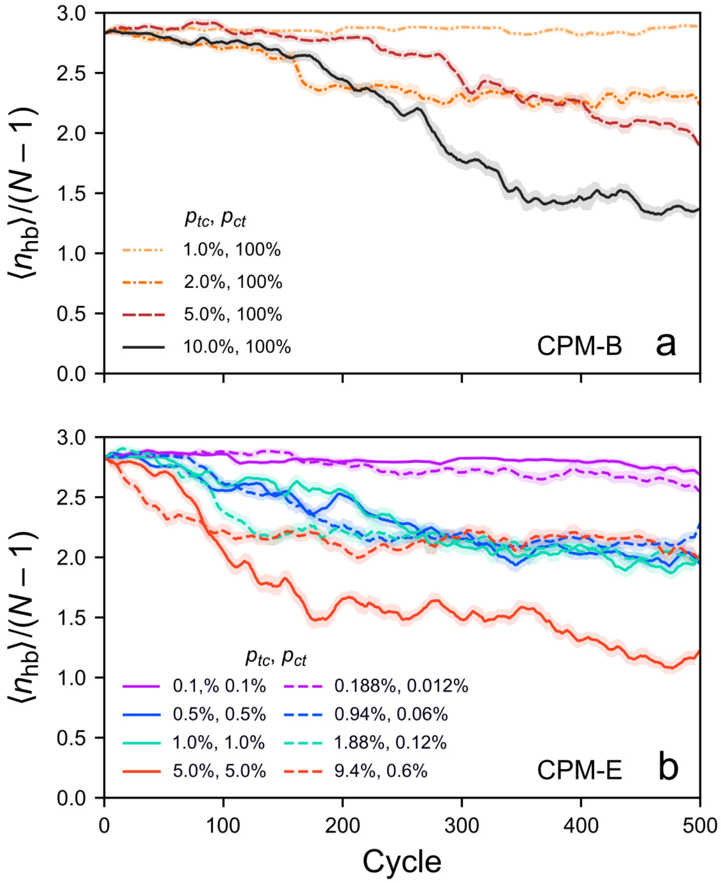

Figure S1: Number of hydrogen bonds per neighboring pair over the course of the CPM simulations. Results of the (**a**) CPM-B and (**b**) CPM-E simulations are shown for different switching probabilities. Each curve is smoothed via a running average (window size of 20 cycles, corresponding to 0.96 ns). Shaded error bands indicate the standard deviation of the raw curve in each window.

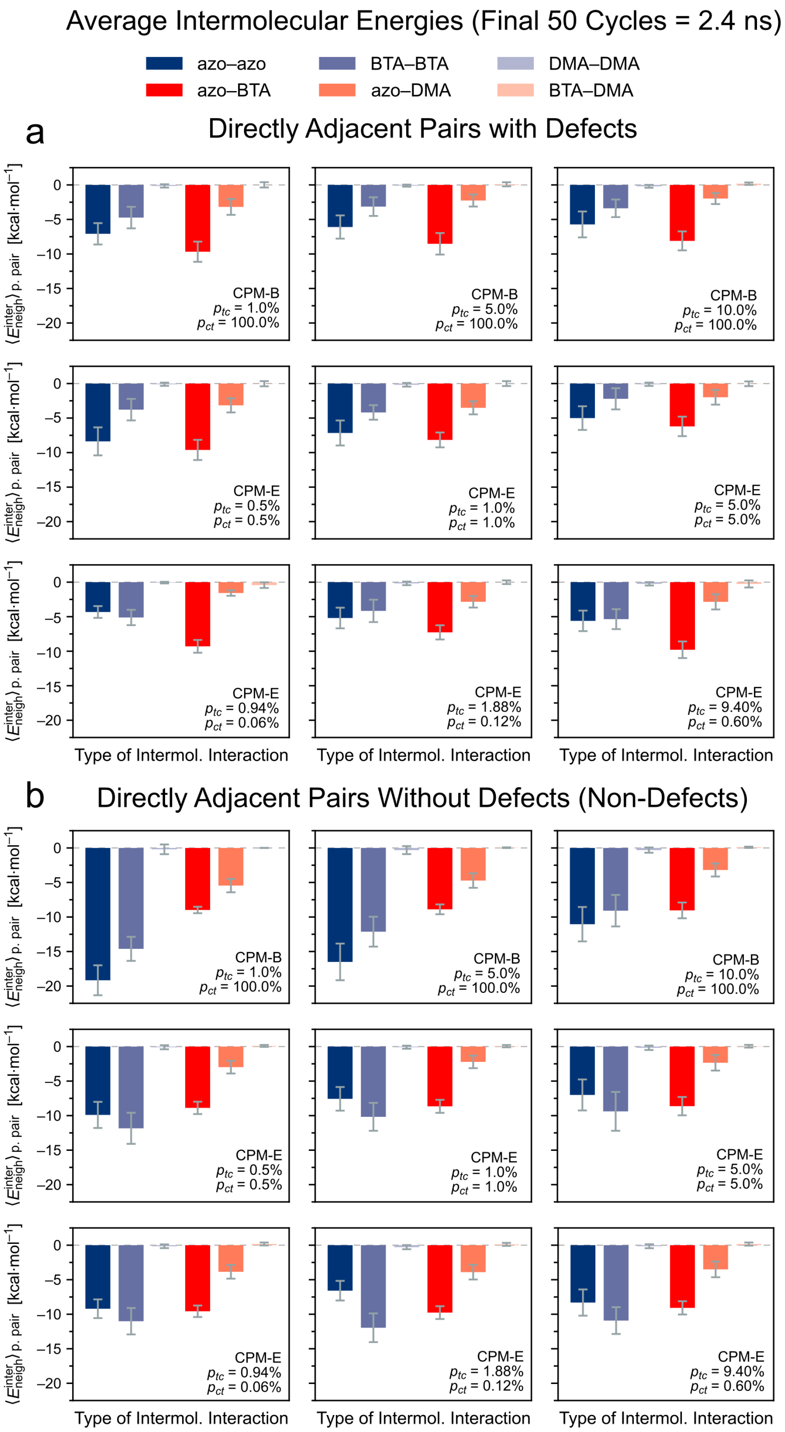

Figure S2: Decomposition of the average intermolecular energy between adjacent pairs of monomers (**a**) with defects and (**b**) without defects during the final 50 cycles (2.4 ns) of the simulations. The decomposition is based on the interactions between the physical parts of the *TrisAzo* molecules. Results of simulation runs with different switching probabilities in the CPM-B and CPM-E approach are shown.

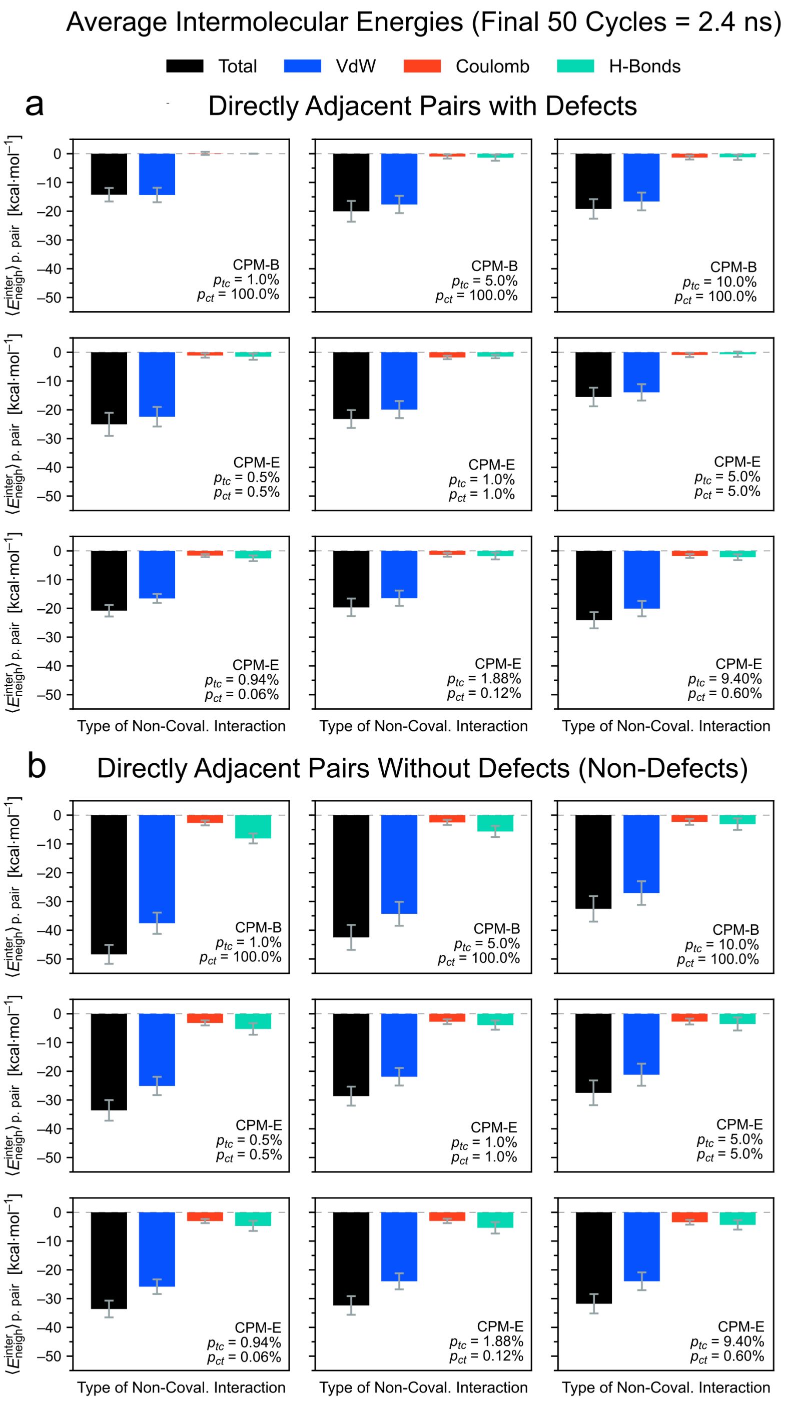

Figure S3: Decomposition of the average intermolecular energy between adjacent pairs of monomers (**a**) with defects and (**b**) without defects during the final 50 cycles (2.4 ns) of the simulations. The decomposition is based on the different types of non-covalent interactions between the *TrisAzo* molecules. Results of simulation runs with different switching probabilities in the CPM-B and CPM-E approach are shown.

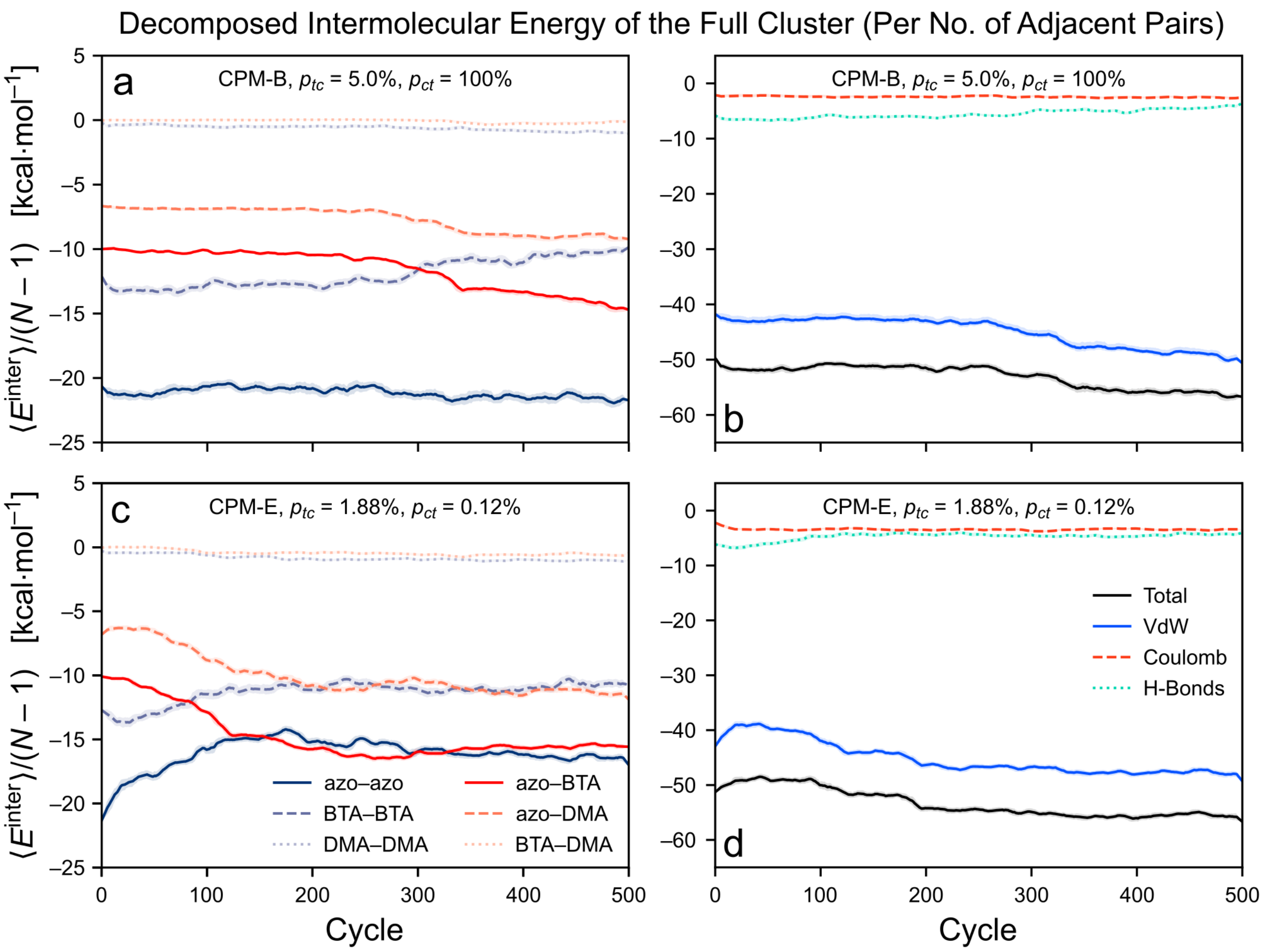

Figure S4: Time evolution of the intermolecular energies of the full *TrisAzo* stack (**a**,**b**) for a CPM-B simulation with $p_{tc} = 5.0\,\%$, and (**c**,**d**) for a CPM-E simulation with $p_{tc} = 1.88\,\%$, $p_{ct} = 0.12\,\%$, Left-hand side: energy decomposition by interactions between the different physical parts of the *TrisAzo* molecules. Right-hand side: energy decomposition by types of non-covalent interactions. All energies are divided by the number of neighboring pairs in the stacks $(N-1)$ for comparability. Each curve is smoothed via a running average (window size of 20 cycles, corresponding to 0.96 ns). Shaded error bands indicate the standard deviation of the raw curve in each window.

S2 Mathematical Model of the Isomer Interconversion

S2.1 *Trans* and *Cis* Isomers of Azobenzene

The fully reversible *trans–cis* photoisomerization of azobenzene may be represented as

$$trans \underset{k_{ct}}{\overset{k_{tc}}{\rightleftharpoons}} cis. \tag{S.1}$$

Here, k_{tc} and k_{tc} are the rate constants for completed interconversions from one isomer type to another. Considering a system of multiple azo moieties, this process can be described by two coupled linear ordinary differential equations (ODE). We assume a fixed number of azobenzene moieties and constant light intensity. The molar fractions x_t and x_c of *trans* and *cis* azobenzene, respectively, then obey the following two equations:

$$\frac{\partial x_t(t)}{\partial t} = -k_{tc}x_t(t) + k_{ct}x_c(t), \qquad \frac{\partial x_c(t)}{\partial t} = +k_{tc}x_t(t) - k_{ct}x_c(t). \tag{S.2}$$

This approach is simplified as compared to the real system to match the simulation approach. A detailed theoretical treatment of the physical system, explicitly modeling the thermally induced back switching of *cis* azobenzene, can be found in Ref. 1. Note that setting either of the two expressions in Equation (S.2) to zero yields the well-known detailed balance condition and corresponds to the system in the photostationary state.

Next, we consider the initial value problem (IVP) with the initial conditions $x_t(t=0) = 1.0$ and $x_c(t=0) = 0.0$, matching the simulations. The solution is obtained straightforwardly by standard methods:

$$x_t(t) = \frac{k_{ct} + k_{tc}\, e^{-(k_{tc}+k_{ct})t}}{k_{tc} + k_{ct}}, \qquad x_c(t) = \frac{k_{tc}\left(1 - e^{-(k_{tc}+k_{ct})t}\right)}{k_{tc} + k_{ct}}. \tag{S.3}$$

For $t \to \infty$, the first derivatives of these functions vanish. Thus, the system reaches the

photostationary state (PSS) with the following molar fractions of the isomers:

$$x_t^{\mathrm{PSS}} = \lim_{t\to\infty} x_t(t) = \frac{k_{ct}}{k_{tc}+k_{ct}}, \qquad x_c^{\mathrm{PSS}} = \lim_{t\to\infty} x_c(t) = \frac{k_{tc}}{k_{tc}+k_{ct}}. \tag{S.4}$$

When the rate constants are defined per photoisomerization cycle – not per time unit – they equal the switching probabilities per cycle, $k_{tc} = p_{tc}$ and $k_{ct} = p_{ct}$. Thus, the expressions in Equation (S.4) are equivalent to those in Equation (2), as expected.

S2.2 Isomers of *TrisAzo*

Similarly, one can describe the time-dependent interconversion of the four *TrisAzo* isomers by four coupled ODE, as shown in the Supporting Information of Ref. 2. We express the model using only parameters that are also inputs to the simulations. The rate constants for the interconversion of the *TrisAzo* isomers are given in terms of the rate constants k_{tc} and k_{tc} of the interconverting azo isomers. For simplicity, we assume that the azo groups isomerize independently of the type of *TrisAzo* isomer they are in and decoupled from the other azo groups in the molecule. Under this condition, the photoisomerization of *TrisAzo* proceeds as

$$ttt \underset{k_{ct}}{\overset{3k_{tc}}{\rightleftharpoons}} ttc \underset{2k_{ct}}{\overset{2k_{tc}}{\rightleftharpoons}} tcc \underset{3k_{ct}}{\overset{k_{tc}}{\rightleftharpoons}} ccc. \tag{S.5}$$

The 3:2:1 ratio of the rate constants results from the number of azo moieties in each type of *TrisAzo* isomer. This ratio is corroborated by the experiments in Ref. 2. Thus, we formulate the following four coupled ODE with constant coefficients:

$$\begin{aligned}
\frac{\partial x_{ttt}(t)}{\partial t} &= -3k_{tc}x_{ttt}(t) + k_{ct}x_{ttc}(t),\\
\frac{\partial x_{ttc}(t)}{\partial t} &= +3k_{tc}x_{ttt}(t) - (2k_{tc}+k_{ct})x_{ttc}(t) + 2k_{ct}x_{tcc}(t),\\
\frac{\partial x_{tcc}(t)}{\partial t} &= +2k_{tc}x_{ttc}(t) - (k_{tc}+2k_{ct})x_{tcc}(t) + 3k_{tc}x_{ccc}(t),\\
\frac{\partial x_{ccc}(t)}{\partial t} &= k_{tc}x_{tcc}(t) - 3k_{tc}x_{ccc}(t).
\end{aligned} \tag{S.6}$$

This may be put into matrix form:

$$\frac{\partial}{\partial t}\begin{pmatrix} x_{ttt}(t) \\ x_{ttc}(t) \\ x_{tcc}(t) \\ x_{ccc}(t) \end{pmatrix} = \begin{pmatrix} -3k_{tc} & k_{ct} & 0 & 0 \\ 3k_{tc} & -(2k_{tc}+k_{ct}) & 2k_{ct} & 0 \\ 0 & 2k_{tc} & -(k_{tc}+2k_{ct}) & 3k_{ct} \\ 0 & 0 & k_{tc} & -3k_{ct} \end{pmatrix} \begin{pmatrix} x_{ttt}(t) \\ x_{ttc}(t) \\ x_{tcc}(t) \\ x_{ccc}(t) \end{pmatrix}. \tag{S.7}$$

We consider the IVP corresponding to the simulations, i.e. $x_{ttt}(t=0) = 1.0$ and $x_{ttc}(t=0) = x_{tcc}(t=0) = x_{ccc}(t=0) = 0.0$. Solving it via standard methods, it results:

$$\begin{aligned} x_{ttt}(t) =& \frac{k_{tc}^3}{(k_{tc}+k_{ct})^3}\left(\frac{k_{ct}^3}{k_{tc}^3} + \frac{3k_{ct}^2}{k_{tc}^2}e^{-(k_{tc}+k_{ct})t} + \frac{3k_{ct}}{k_{tc}}e^{-2(k_{tc}+k_{ct})t} + e^{-3(k_{tc}+k_{ct})t}\right), \\ x_{ttc}(t) =& \frac{k_{tc}^3}{(k_{tc}+k_{ct})^3}\left(\frac{3k_{ct}^2}{k_{tc}^2} + \frac{3(2k_{tc}k_{ct}-k_{ct}^2)}{k_{tc}^2}e^{-(k_{tc}+k_{ct})t} + \right. \\ & \left. \frac{3(k_{tc}-2k_{ct})}{k_{tc}}e^{-2(k_{tc}+k_{ct})t} - 3e^{-3(k_{tc}+k_{ct})t}\right), \\ x_{tcc}(t) =& \frac{k_{tc}^3}{(k_{tc}+k_{ct})^3}\left(\frac{3k_{ct}}{k_{tc}} + \frac{3(k_{tc}-2k_{ct})}{k_{tc}}e^{-(k_{tc}+k_{ct})t} + \right. \\ & \left. \frac{3(k_{ct}-2k_{tc})}{k_{tc}}e^{-2(k_{tc}+k_{ct})t} + 3e^{-3(k_{tc}+k_{ct})t}\right), \\ x_{ccc}(t) =& \frac{k_{tc}^3}{(k_{tc}+k_{ct})^3}\left(1 - 3e^{-(k_{tc}+k_{ct})t} + 3e^{-2(k_{tc}+k_{ct})t} - e^{-3(k_{tc}+k_{ct})t}\right). \end{aligned} \tag{S.8}$$

For $t \to \infty$ the first derivatives of these functions vanish. Thus, the PSS is reached with

$$\begin{aligned} x_{ttt}^{\mathrm{PSS}} &= \lim_{t\to\infty} x_{ttt}(t) = \frac{k_{ct}^3}{(k_{tc}+k_{ct})^3}, \\ x_{ttc}^{\mathrm{PSS}} &= \lim_{t\to\infty} x_{ttc}(t) = \frac{3k_{tc}k_{ct}^2}{(k_{tc}+k_{ct})^3}, \\ x_{tcc}^{\mathrm{PSS}} &= \lim_{t\to\infty} x_{tcc}(t) = \frac{3k_{tc}^2k_{ct}}{(k_{tc}+k_{ct})^3}, \\ x_{ccc}^{\mathrm{PSS}} &= \lim_{t\to\infty} x_{ccc}(t) = \frac{k_{tc}^3}{(k_{tc}+k_{ct})^3}. \end{aligned} \tag{S.9}$$

These molar fractions of the *TrisAzo* isomers correspond to Equation (3), which was obtained via statistical considerations. Note that the rate constants $k_{tc/ct}$ are directly proportional to the switching probabilities $p_{tc/ct}$.

S3 Descriptors of the Cluster Structure

The here introduced descriptors are used to characterize the structure of the *TrisAzo* stack. The description below is based on the thesis of M.K.[3]

S3.1 Definition of Observables and Order Parameters

Stacking Distances

The stacking distance or pairwise distance $\Delta r_i(t)$ at time t is defined as the absolute distance between the centers of mass of two adjacent *TrisAzo* molecules (pair index i) in the stack. Its time average is $\langle \Delta r_i \rangle_t$. Averaging $\Delta r_i(t)$ over all adjacent pairs yields $\langle \Delta r(t) \rangle_{N-1} \equiv \Delta r(t)$ and additionally time-averaging yields $\langle \Delta r \rangle_t$.

Local Columnar Orientation Order Parameter

The orientational order of the monomers in the columnar cluster is evaluated via the inclination angles $\Delta\alpha_i(t)$ between the adjacent *TrisAzo* molecules with pair index i. The inclination angles are obtained by first computing the orientation unit vectors $\vec{u}_i(t)$ and $\vec{u}_{i+1}(t)$, which are pointing along the shortest axes of the disk-like *TrisAzo* molecules i and $i+1$. For each molecule, the gyration tensor is diagonalized and the eigenvalue that corresponds to the smallest eigenvalue is selected, normalized and set equal to $\vec{u}_i(t)$ (compare Ref. 4). For the simulations involving the photoisomerization of the azo groups, the inclination angles are computed between the phenyl rings at the very center of each *TrisAzo* molecule. These parts remain always flat and oblate, even when the azo moieties switch to *cis*, thereby changing the overall shape of the molecules.

Using the inclination angles, we compute the so-called local columnar orientation order parameter $\Psi_i(t)$:

$$\Psi_i(t) = |\vec{u}_i(t) \cdot \vec{u}_{i+1}(t)| = |\cos \Delta\alpha_i(t)| \,. \tag{S.10}$$

The parameter $\Psi_i(t)$ is a local measure of orientational alignment between two monomers i and $i+1$ (monomers pair with pair index i). Its time average is $\langle \Psi_i \rangle_t$.

Global Columnar Orientation Order Parameter

Averaging $\Psi_i(t)$ over all $N-1$ adjacent pairs in a stack with N monomers yields $\langle \Psi(t) \rangle_{N-1} \equiv \Psi(t)$, the so-called global columnar orientation order parameter:

$$\Psi(t) = \frac{1}{N-1} \sum_{i=1}^{N-1} |\vec{u}_i(t) \cdot \vec{u}_{i+1}(t)| = \frac{1}{N-1} \sum_{i=1}^{N-1} |\cos \Delta\alpha_i(t)| \,. \tag{S.11}$$

This parameter is a measure of the columnar shape of the full stack of molecules at a given time t. Its time average is $\langle \Psi \rangle_t$.

Note that due to the oblate shape of *TrisAzo* (or, alternatively, its central phenyl ring), flipping such a molecule by 180° yields the same orientation. Thus, by taking the absolute values in Equations (S.10) and (S.11), the orientation vectors $\vec{u}_i(t)$ and $-\vec{u}_i(t)$ are treated as equivalents. Therefore, an ideal columnar stack corresponds to $\Psi(t) = 1$. Clusters with randomly oriented molecules yield $\Psi(t) \approx 0.5$.

S3.2 Defect Detection Algorithm

We determine the position and number of defects in *TrisAzo* clusters based on absolute criteria to ensure reproducibility. For the full MD trajectory, we compute the time averages $\langle \dots \rangle_t$ and standard deviations σ of the stacking distances, $\Delta r_i(t)$, and columnar orientation order parameters, $\Phi_i(t)$, of all adjacent monomer pairs i in the stack. A monomer pair is regarded as a "defect" if at minimum one of the following four criteria is satisfied, or as a "non-defect" in the opposite case:

- $\langle \Delta r_i \rangle_t > 6.0\,\text{Å}$,
- $\sigma(\Delta r_i) > 2.0\,\text{Å}$,
- $\langle \Phi_i \rangle_t < 0.5$,
- $\sigma(\Phi_i) > 0.35$.

These criteria have been determined by matching the results of visual defect detection with the results of the defect detection algorithm for selected MD trajectories.

References

(1) Toshchevikov, V.; Ilnytskyi, J.; Saphiannikova, M. Photoisomerization Kinetics and Mechanical Stress in Azobenzene-Containing Materials. *J. Phys. Chem. Lett.* **2017**, *8*, 1094–1098, DOI: `10.1021/acs.jpclett.7b00173`.

(2) Kind, J.; Kaltschnee, L.; Leyendecker, M.; Thiele, C. M. Distinction of *trans–cis* photoisomers with comparable optical properties in multiple-state photochromic systems – examining a molecule with three azobenzenes *via in situ* irradiation NMR spectroscopy. *Chem. Commun.* **2016**, *52*, 12506–12509, DOI: `10.1039/C6CC06771A`.

(3) Koch, M. The Influence of Light on a Three-Arm Azobenzene Star: A Computational Study. Ph.D. Dissertation, Technische Universität Dresden, 2022.

(4) Ilnytskyi, J. M.; Toshchevikov, V.; Saphiannikova, M. Modeling of the photo-induced stress in azobenzene polymers by combining theory and computer simulations. *Soft Matter* **2019**, *15*, 9894–9908, DOI: `10.1039/C9SM01853K`.
